# Prevention of Fetal/Neonatal Alloimmune Thrombocytopenia in Mice: Biochemical and Cell Biological Characterization of Isoforms of a Human Monoclonal Antibody

**DOI:** 10.4049/immunohorizons.2100097

**Published:** 2022-01-24

**Authors:** Trude V. Mørtberg, Huiying Zhi, Gestur Vidarsson, Stian Foss, Suzanne Lissenberg-Thunnissen, Manfred Wuhrer, Terje E. Michaelsen, Bjørn Skogen, Tor B. Stuge, Jan Terje Andersen, Peter J. Newman, Maria Therese Ahlen

**Affiliations:** * Norwegian National Unit for Platelet Immunology, Division of Diagnostics, Department of Laboratory Medicine, University Hospital of North Norway, Tromsø, Norway; † Department of Medical Biology, UiT The Arctic University of Norway, Tromsø, Norway; ‡ Blood Research Institute, Versiti Blood Center of Wisconsin, Milwaukee, WI; § Department of Experimental Immunohematology, Sanquin Research and Landsteiner Laboratory, Amsterdam University Medical Center, University of Amsterdam, Amsterdam, the Netherlands; ¶ Department of Immunology, Oslo University Hospital Rikshospitalet, University of Oslo, Oslo, Norway; ║ Department of Pharmacology, Institute of Clinical Medicine, University of Oslo, Oslo, Norway; # Center for Proteomics and Metabolomics, Leiden University Medical Center, Leiden, the Netherlands; ** Department of Infection Immunology, Norwegian Institute of Public Health, Oslo, Norway; †† School of Pharmacy, University of Oslo, Oslo, Norway

## Abstract

Maternal alloantibodies toward paternally inherited Ags on fetal platelets can cause thrombocytopenia and bleeding complications in the fetus or neonate, referred to as fetal and neonatal alloimmune thrombocytopenia (FNAIT). This is most commonly caused by Abs against the human platelet Ag (HPA)-1a in Caucasians, and a prophylactic regimen to reduce the risk for alloimmunization to women at risk would be beneficial. We therefore aimed to examine the prophylactic potential of a fully human anti–HPA-1a IgG1 (mAb 26.4) with modified Fc region or altered N-glycan structures. The mAb 26.4 wild-type (WT) variants all showed efficient platelet clearance capacity and ability to mediate phagocytosis independent of their N-glycan structure, compared with an effector silent variant (26.4.AAAG), although the modified N-glycan variants showed differential binding to FcγRs measured in vitro. In an in vivo model, female mice were transfused with platelets from transgenic mice harboring an engineered integrin β3 containing the HPA-1a epitope. When these preimmunized mice were bred with transgenic males, Abs against the introduced epitope induced thrombocytopenia in the offspring, mimicking FNAIT. Prophylactic administration of the mAb 26.4.WT, and to some extent the mAb 26.4.AAAG, prior to platelet transfusion resulted in reduced alloimmunization in challenged mice and normal platelet counts in neonates. The notion that the effector silent variant hampered alloimmunization demonstrates that rapid platelet clearance, as seen with mAb 26.4.WT, is not the sole mechanism in action. Our data thus successfully demonstrate efficient Ab-mediated immunosuppression and prevention of FNAIT by anti–HPA-1a monoclonal variants, providing support for potential use in humans.

## INTRODUCTION

Fetal and neonatal alloimmune thrombocytopenia (FNAIT) is a clinical condition caused by generation of maternal IgG Abs directed toward alloantigens on fetal platelets. The human platelet Ag (HPA) most commonly associated with FNAIT in Caucasians is HPA-1a (1). Women lacking this Ag may become alloimmunized when encountering it during pregnancy or in connection with delivery. The maternal IgG Abs are then transferred over the placenta and enter the fetal circulation. At this point, the IgG Abs bind HPA-1a followed by induction of platelet depletion, which renders the fetus prone to bleeding, with the risk for intracranial hemorrhage and lifelong disabilities or, in the worst case, death (2). Most identified cases are recognized after delivery because of bleeding symptoms in the neonate, and the condition is thus underdiagnosed (3). Unfortunately, no preventive or effective treatment for FNAIT is available, but the off-label use of IVIg to treat immunized women is recommended in several clinical guidelines (4).

Alloimmunization may occur during pregnancy; however, data from a prospective screening study in Norway showed that up to 75% of the women were immunized in connection with delivery (5). This opens the possibility for prophylactic treatment to prevent HPA-1a alloimmunization, in line with administration of anti-D IgG prophylaxis that has safely and efficiently prevented alloimmunization to RhD Ag, which has reduced the incidence of hemolytic disease of the fetus and newborn in the past 50 y (6). This notion motivated the development of the hyperimmune human plasma-derived anti-HPA-1a IgG preparation named NAITgam (also referred to as RLYB211), which is currently undergoing clinical trials to evaluate its ability to reduce the incidence of HPA-1a immunizations in HPA-1a-negative pregnant women (7). An even more attractive strategy is the use of a monoclonal IgG Ab specific for HPA-1a as a prophylaxis, because this will allow streamlined manufacturing of a product with a well-defined mechanism of action without the need for plasma supply from immunized individuals.

To develop prophylactic treatment for FNAIT, it may be worthwhile to look into experiences gained on anti-D prophylaxis for hemolytic disease of the fetus and newborn. However, despite decades of research and clinical studies, recombinant monoclonal anti-D prophylactic products have not yet proven to be effective substitutes for the polyclonal products on the market (8). The reason for this is not known, although it could be related to the size of the D-Ag, which could harbor multiple potential epitopes. In contrast, HPA-1a is defined by a single amino acid difference between mother and child (1, 9). Thus, a single HPA-1a-specific Ab will in principle sterically block the entire alloantigenic epitope when bound. The use of prophylactic Abs to prevent alloimmunization is based on the concept of Ab-mediated immune suppression (AMIS) (10), but optimal design for mAbs directed against HPA-1a has not yet been defined.

In this study, we made use of the human anti-HPA-1a, mAb 26.4, derived from a woman who gave birth to a child with severe FNAIT (11). This mAb has been shown to be highly specific to the HPA-1a epitope on integrin β3. Here, we explored it as a prophylactic candidate against HPA-1a immunization by a combination of in vitro and in vivo tests. To do so, we engineered a panel of recombinant IgG1 variants of mAb 26.4 where we introduced specific Fc-region amino acid substitutions or altered the composition of attached N-glycan structures. The panel of isoforms was studied for their ability to interact with recombinant effector molecules, including the plasma half-life regulator FcRn, combined with cellular studies addressing phagocytosis and platelet clearance.

For in vivo studies, we used a recently developed humanized FNAIT preclinical mouse model (12), where five amino acid substitutions (APLDQ) have been engineered into the murine integrin β3, which mimics the human HPA-1a epitope and thus allowing for mAb 26.4 binding. We demonstrate that the mAb 26.4 wild-type (WT) is able to induce rapid platelet clearance and prevent alloimmunization and subsequent FNAIT symptoms in offspring. Notably, an engineered mAb 26.4 variant lacking effector functions also showed dampened immunization, although it was not capable of inducing platelet clearance. These results support further preclinical and clinical studies of mAb 26.4 as a prophylactic drug to prevent HPA-1a alloimmunization in humans.

## MATERIALS AND METHODS

### Ab production and verification

Vectors encoding recombinant mAb 26.4 isoforms were produced as previously described (13, 14). Briefly, cDNA fragments encoding the 26.4 variable H and L chain regions were codon optimized for mammalian expression and subcloned in-frame with the cDNA sequence of the human IgG1 constant H and L chain regions in pcDNA3.1 expression vectors. In addition to the mAb 26.4.WT, the effector silent variant 26.4.AAAG (L234A, L235A, N297A, P329G) and 26.4.H435A with reduced FcRn binding (15, 16) were produced. mAb 26.4.WT was also produced containing altered N-glycans, low fucose (LF; 26.4.WT.LF), and high galactose (HG; 26.4.WT.HG). The mAb variants were produced using FreeStyle 293-F cells (Thermo Fisher Scientific, Waltham, MA). Variants with modified N297-linked N-glycans were produced by supplying a fucose decoy, 2-deoxy-2-fluoro-L-fucose, posttransfection to decrease fucosylation, or by adding 5 mM D-galactose (Sigma-Aldrich, Burlington, MA) and expression vector encoding β−1,4-galactosyltransferase 1 to the transfection mix to increase galactosylation. The mAb variants were purified using a Protein A HiTrap HP affinity column (GE Healthcare, Chicago, IL), and their integrity was verified by SDS-PAGE. Fc glycosylation, including galactosylation and fucosylation, was assessed by mass spectrometry.

### Ag production and verification

To verify Ab characteristics, we produced recombinant soluble integrin β3 proteins using the baculovirus system (17, 18). Originally designed constructs for the extracellular domain murine integrin β3 (synthesized GeneArt; Thermo Fisher Scientific) were codon optimized for *Spodoptera frugiperda* with XhoI and NheI restriction sites flanking the insert for cloning. Because some construct changes were necessary for this study, tags and epitopes were changed using the GeneArt Site-Directed Mutagenesis System (Thermo Fisher Scientific). Optimized constructs contained a kozak sequence at the N terminus, and a 6xHis and a Twin-Strep-tag (WSHPQFEK-GGGSGGGSGGS-SAWSHPQFEK), followed by two stop codons, at the C terminus. The constructs were first moved into the acceptor vector pFL (gift from EMBL) followed by transformation into EMBacY (19) expressing *Escherichia coli* cells (gift from EMBL) through Tn7 transposition (18). Cells with correctly inserted pFL vector were chosen through blue/white selection. Bacmids were purified by isopropanol precipitation from subcultured EMBacY-expressing *E. coli* cells and used for transfection of Sf9 insect cells (Thermo Fisher Scientific) for baculovirus production. Proteins were expressed in H5 insect cells (Thermo Fisher Scientific) using titered viral stocks with daily monitoring of the coproduced YFP protein by flow cytometry.

After dialysis and concentration of the supernatant using the polyethersulfone VivaFlow 200 system, 30,000 molecular weight cutoff (Sartorius, Goöttingen, Germany), the proteins were purified by gravity flow using columns with Strep-Tactin XT Superflow resin (IBA Lifesciences, Goöttingen, Germany). The presence and purity of recombinant proteins were verified by nonreducing SDS-PAGE. Proteins were coupled to Dynabeads His-Tag Isolation & Pulldown (Thermo Fisher Scientific) according to protocol and analyzed using anti-integrin β3 Abs (mAb 26.4 and AP-3, both conjugated to Alexa Fluor 488) by flow cytometry.

### ELISA

#### Binding of mAb 26.4 variants to recombinant mouse integrin β3-APLDQ.

Ninety-six-well enzyme immunoassay (EIA)/RIA plates (CorningCostar) were coated overnight (4°C) with Ag (1 mg/ml in PBS) and blocked with PBS/4% skimmed milk powder (S) (Acumedia)/0.05% Tween 20 (T) (Sigma-Aldrich) (PBS/S/T). Titration series of the mAb 26.4 variants (1.0 to 0.008 mg/ml) in PBS/S/T were added for 1 h at room temperature (RT). Bound Abs were detected using an alkaline phosphatase (AP)-conjugated goat anti-human κ L chain Ab (1:5000; Sigma-Aldrich) and visualized by addition of phosphatase substrate (10 mg/ml in diethanolamine) (Sigma-Aldrich) before absorbance values (405 nm) were recorded using a Sunrise plate reader (TECAN). PBS/T was used as washing buffer in between each step.

#### Binding of mAb 26.4 variants to recombinant human FcγR.

Ninety-six-well EIA/RIA plates were coated with mouse integrin β3-APLDQ (1 μg/ml PBS) and blocked as described earlier, prior to incubation with titration series of the mAb 26.4 variants (2.0 to 0.016 or 1.0 to 0.00005 μg/ml, PBS/S/T) for 1 h at RT. Then, soluble recombinant GST-tagged FcγRI (0.25 μg/ml), FcγRIIa-R131 (2 μg/ml), FcγRIIb (2 μg/ml), or FcγRIIIa-V158 (2 μg/ml) (20) was added and incubated for 1 h at RT before bound receptors were detected using an HRP-conjugated goat anti-GST Ab (1:5000, PBS/S/T; Rockland Immunochemicals, Limerick, PA). Binding was visualized by addition of TMB solution (Calbiochem), and the enzymatic reaction was stopped with 50 μl 1 M HCl before absorption values (450 nm) were recorded as described earlier. PBS/T was used as washing buffer in between each step.

#### Binding of mAb 26.4 variants to recombinant mouse and human FcRn.

Ninety-six-well EIA/RIA plates were coated with mouse integrin β3-APLDQ (1 μg/ml PBS) and blocked as described earlier, prior to incubation with titration series of mAb 26.4 variants (1.0 to 0.008 μg/ml, PBS/S/T) for 1 h at RT. All remaining steps were performed using phosphate buffer (pH 6.0; 67 mM phosphate, 0.1 M NaCl, 0.05% S/T) or PBS/S/T (pH 7.4) as dilution and wash buffers. Recombinant soluble mouse or human GST-tagged FcRn (21, 22) (1 μg/ml) was added and incubated for 1 h at RT. Bound receptors were detected using an HRP-conjugated anti-GST Ab as described earlier, before absorption values (450 nm) were recorded.

### In vivo plasma half-life

Hemizygous FcRn [B6.Cg-*FcgRt*^*tm1Dcr*^ Tg(FCΓRT)32Dcr/DcrJ] mice (JAX stock no. 014565), which are knockout for mouse FcRn HC (*FcgRt*^*tm1Dcr*^) and express the genomic transgene of the human FcRn HC (FCΓRT) under the control of the human FcRn promoter (Tg32), were used to determine plasma half-life. Male mice aged 7–9 wk, weighing between 17 and 30 g, received 1 mg/kg mAb 26.4 variants by i.v. administration. Blood samples (25 μl) were drawn from the retro-orbital sinus at days 1, 2, 3, 5, 7, 10, 12, 16, and 19 postadministration and were mixed with 1 ml 1% K3-EDTA to prevent coagulation followed by centrifugation at 17,000 × *g* for 5 min at 4°C. Plasma was isolated and diluted 1:10 in 50% glycerol/PBS solution and stored at −20°C. Half-life data were plotted as percent of Ab remaining compared with the first concentration measured. Data points from the β-phase were used to calculate half-life using the following formula:

$$t\frac{1}{2}=\frac{\log(0.5)}{\log\left(\frac{A_{c}}{A_{o}}\right)}\times t,$$

where *t*_1/2_ is the half-life of the Ab, *A*_c_ is the amount of Ab remaining, *A*_o_ is the original amount of Ab at day 1, and *t* is the elapsed time (23). The experiment was performed by JAX Services at The Jackson Laboratory, and samples were shipped for analyses.

### Quantification of 26.4 mAb variants in plasma

26.4 mAb variants in plasma were captured on a mouse anti-human IgG Fc mAb (1 mg/ml) (Southern Biotechnology, Birmingham, AL) coated in 96-well EIA/RIA plates (CorningCostar) detected by an AP-conjugated polyclonal goat anti-human κ L chain Ab (Southern Biotechnology) (1:5000), which was visualized by adding the AP substrate (10 μg/ml in diethanolamine) (Sigma-Aldrich). Absorbance was measured at 405 nm and recorded as described earlier. PBS/T was used as washing buffer in between each step.

### Phagocytosis assay using THP-1 cells

FluoSpheres Carboxylate-Modified Microspheres (1.0 μm yellow-green fluorescent; Thermo Fisher Scientific) were coupled to recombinant murine integrin β3-APLDQ protein according to protocol. Briefly, proteins and microspheres were incubated overnight at RT in the presence of 1-ethyl-3-(3-dimethylaminopropyl)-carbodiimide at pH 6.5. After removal of unbound protein, beads were blocked with BlockAid solution (Thermo Fisher Scientific) and stored at 4°C. THP-1 (ATCC TIB-202) cells were cultured at 37°C and 5% CO_2_ in ATCC-modified RPMI 1640 medium (Thermo Fisher Scientific), 0.05 mM 2-ME, and 10% FBS.

The phagocytosis assay protocol was optimized from Ackerman et al. (24). In a 96-well U-bottom plate, 10 μl Fluospheres at ~3.6 × 10^8^/ml were incubated with 10 μl 26.4 Ab (20 μg/ml) for 2 h at 37°C. After washing, beads were resuspended by supplying 200 μl THP-1 cells at 2.5 × 10^5^/ml. The plate was incubated overnight at 37°C and 5% CO_2_. A total of 100 μl supernatant was replaced with 100 μl 4% paraformaldehyde before analyzing the cells using high-throughput screening on BD LSRFortessa (BD Biosciences, San Jose, CA). The gate was set around live THP-1 cells, and 10,000 events were collected. Integrated mean fluorescence intensity (iMFI) was calculated by multiplying the percentage of bead-positive cells with the mean fluorescence intensity of bead-positive cells (24). 26.4.WT and 26.4.AAAG were incubated with APLDQ-coated FluoSpheres as described earlier, followed by incubation with THP-1 cells for 1 h. The cells were analyzed by Amnis ImageStreamX Mk II (Luminex, Austin, TX) using 488 laser 0.3 mW and ×60 focus. The data were further analyzed using the IDEAS software and Internalization Wizard.

### Mice

BALB/c WT female mice (6–10 wk) and C57BL/6N mice (both sexes; 8 wk) were purchased from Charles River. C57BL/6N-APLDQ mice (in-house) were mainly used for blood platelet isolation (both sexes; >8 wk of age). Mice were maintained in the Biological Resource Center at the Medical College of Wisconsin.

All animal protocols were approved by the Medical College of Wisconsin Institutional Animal Care and Use Committee and performed according to the human and animal experimentation guidelines of the U.S. Department of Health and Human Services and in adherence to the National Institutes of Health *Guide for the Care and Use of Laboratory Animals*.

### Blood sampling

Blood from adult mice was sampled using EDTA-coated capillary tubes from the submandibular vein. Platelet count was measured using a ScilVet ABC (scil Animal Care Company, Gurnee, IL). Samples were diluted 1:3 in PBS with 0.3% EDTA and collected after centrifugation at 700 × *g* for 15 min. Blood from pups was collected within 48 h of delivery from the submandibular vein using heparinized microhematocrit capillary tubes (Thermo Fisher Scientific) and transferred to tubes with anticoagulant acid-citrate-dextrose. All pups were euthanized directly after blood collection. Samples were diluted 1:3 in PBS with 0.3% EDTA and collected after centrifugation at 700 × *g* for 15 min. All samples were stored at −70°C until use.

### Platelet isolation, immunization, and prophylaxis

APLDQ mice were anesthetized using a ketamine mix (20 mg/ml ketamine and 2 mg/ml xylazine) 0.5 ml/20 g body weight injected i.p. When deep anesthesia was reached, the mouse was strained and opened, exposing the posterior vena cava. Blood was drawn slowly into syringes prefilled with anti-coagulant acid-citrate-dextrose. Death was assured by cervical dislocation. Blood collected from the APLDQ mice was pooled, diluted 1:2 in PBS, and layered on top of Fico/Lite-LM (Atlanta Biologicals, Flowery Branch, GA). The tubes were centrifuged at 350 × *g* for 15 min without brakes. Platelet-rich plasma was recovered, and the platelet count was measured as described earlier. Washed platelets were resuspended to 5 × 10^8^/ml in PBS. Platelets (1 × 10^8^/dose) were injected i.v. into female BALB/c mice to be immunized (days 2 and 9). 26.4 Abs were diluted in PBS, 20 μg Ab/200 μl, and injected i.v. 24 h prior to APLDQ platelet transfusion for prophylactic experiments (days 1 and 8).

### Breeding

At least 3 d after the last blood collection, BALB/c females from the prophylaxis experiment were placed in separate cages and introduced to APLDQ males. Within 48 h after delivery, pups were weighed, and blood samples were drawn as described earlier. Both blood platelet count and the presence of anti-APLDQ Abs in plasma were analyzed.

### Plasma analysis

#### Abs against mouse platelets.

To analyze Abs in plasma from immunized mice or pups, 96-well U-bottom plates were blocked with 1% PBSA for 1 h. 1 × 10^6^ platelets from APLDQ mice or control mice and plasma samples (end dilution 1:6 to test for mouse anti-APLDQ Abs and 1:12 to test for 26.4 Abs), were incubated for 30 min at RT. FITC-conjugated anti-mouse IgG or anti-human IgG (Jackson ImmunoResearch, Philadelphia, PA) diluted 1:200 was incubated for 30 min at RT, and Ab binding was measured using BD Accuri C6 (BD Biosciences). A total of 10,000 events were collected from the platelet gate.

#### Mouse anti-human Abs.

26.4.WT Abs were coupled to Dynabeads M-270 Epoxy (Thermo Fisher Scientific) following Ab-coupling protocol. Briefly, washed beads were incubated with Ab on a roller at 37°C overnight. Washed beads were stored at 4°C until use.

Ab-coated beads (500,000 beads/well) were incubated with murine plasma (end dilution 1:200) by shaking for 20 min. Mouse anti-human Abs (MAHAs) in plasma were detected using anti-mouse IgG conjugated with Alexa Fluor 647 (1:500; Jackson Immunoresearch), and beads were analyzed using high-throughput screening on BD LSRFortessa (BD Biosciences).

### Statistical analysis

ELISA data are plotted as mean ± SD. Flow cytometric data are plotted with median fluorescence intensity (MFI) and presented as mean ± SD. Statistical analyses were done using GraphPad Prism 9.2.0 software by ordinary one-way ANOVA and Tukey multiple comparison test (*p ≤ 0.05, **p ≤ 0.01, ***p ≤ 0.001, and ****p ≤ 0.0001).

## RESULTS

### IgG1 mAb 26.4 variants engineered for distinct effector molecule binding properties

**FIGURE 1.**
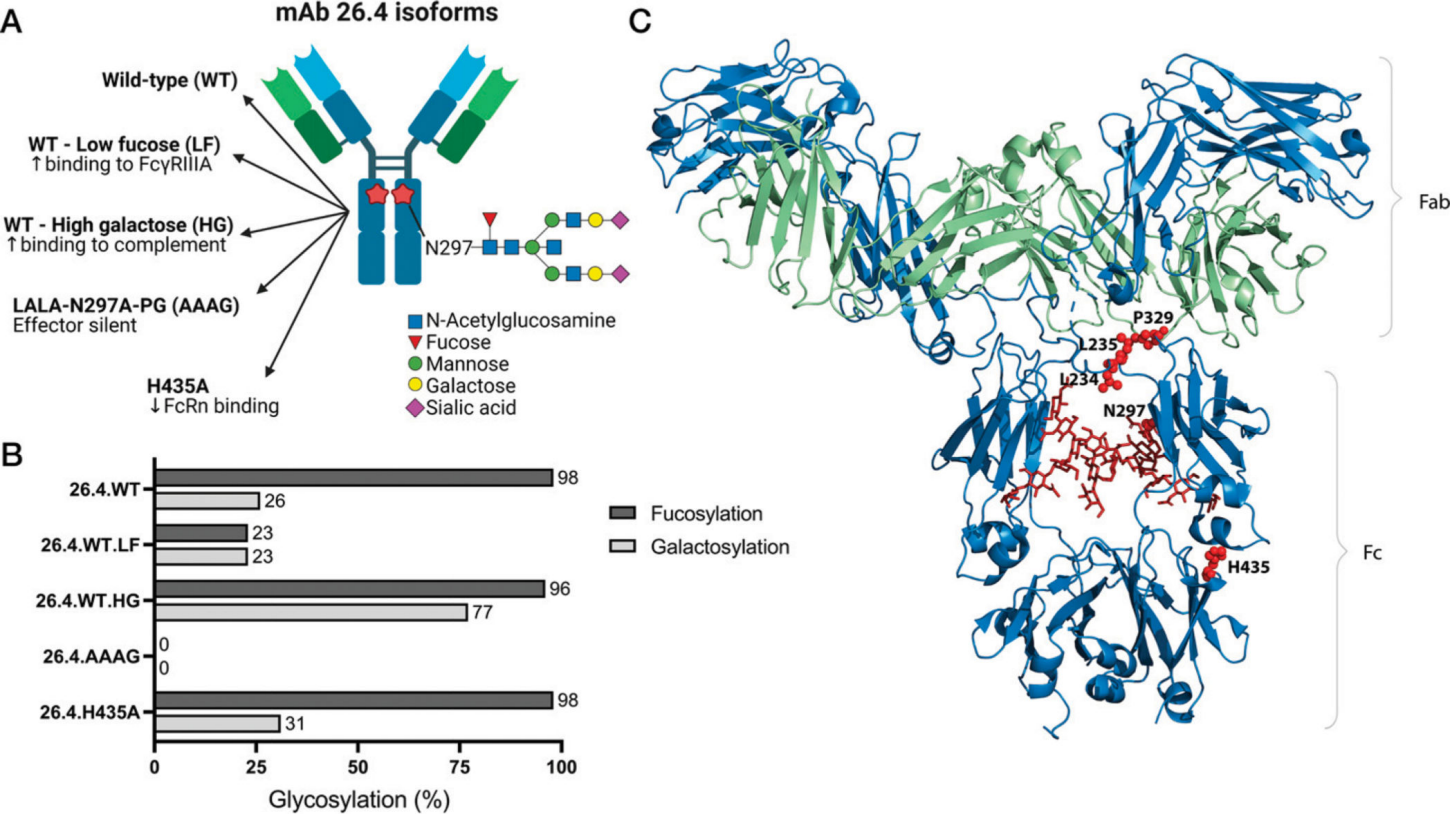
Design of anti–HPA-1a mAb 26.4 isoforms with different Fc regions. (**A**) Schematic illustration of a monoclonal anti-HPA-1a IgG1 26.4 Ab with different modifications highlighted (created with BioRender.com). (**B**) The N-glycan structures attached to N297 of recombinantly produced 26.4 variants were analyzed by mass spectrometry to map the content of fucose and galactose. (**C**) Illustration showing the crystal structure of a human IgG1 molecule. The heavy chains are shown in blue, light chains in green, and N297-linked N-glycans as red sticks. The amino acid residues L234, L235, P329, N297, and H435 are shown as red spheres. The figure was made in PyMOL using the crystallographic data from PDB: 1HZH. AAAG, LALA-N297A-PG.

The preferred effector function properties of a prophylactic mAb to prevent HPA-1a immunization should be tailored prior to clinical testing in humans, and the requirement may vary depending on the treatment regimen. Thus, to study the effect of modified Fc-mediated effector functions, we engineered a panel of IgG1 isoforms based on mAb 26.4 (Fig. 1A). To completely abolish the ability of WT IgG1 mAb 26.4 (26.4.WT) to engage all classical human FcγRs and the complement factor C1q, we introduced four amino acid substitutions in the CH2 domain of the H chain, namely, L234A, L235A, N297A, and P329G (AAAG) (25). Notably, N297 is the only N-glycan site in the Fc, and distinct biantennary glycan structures may modulate binding to individual effector molecules (26). As such, we produced recombinant 26.4.WT variants with LF (23% fucose versus 98% in WT) and HG (77% galactose versus 26% in WT) content (Fig. 1B), which enhances binding to FcγRIIIa on NK cells (27) and binding to C1q, respectively (28).

The designed mAbs were produced by FreeStyle 293-F cells, and secreted Abs were purified with expected molecular size as determined by SDS-PAGE analysis (Supplemental Fig. 1A).

To confirm functional binding to human integrin β3, we produced recombinant soluble proteins. Size and purity were confirmed by SDS-PAGE analysis (Supplemental Fig. 2A), and appropriate epitope conformation was confirmed by reactivity with anti-integrin β3 mAbs (AP-3 and 26.4) in flow cytometry (Supplemental Fig. 2B).

**FIGURE 2.**
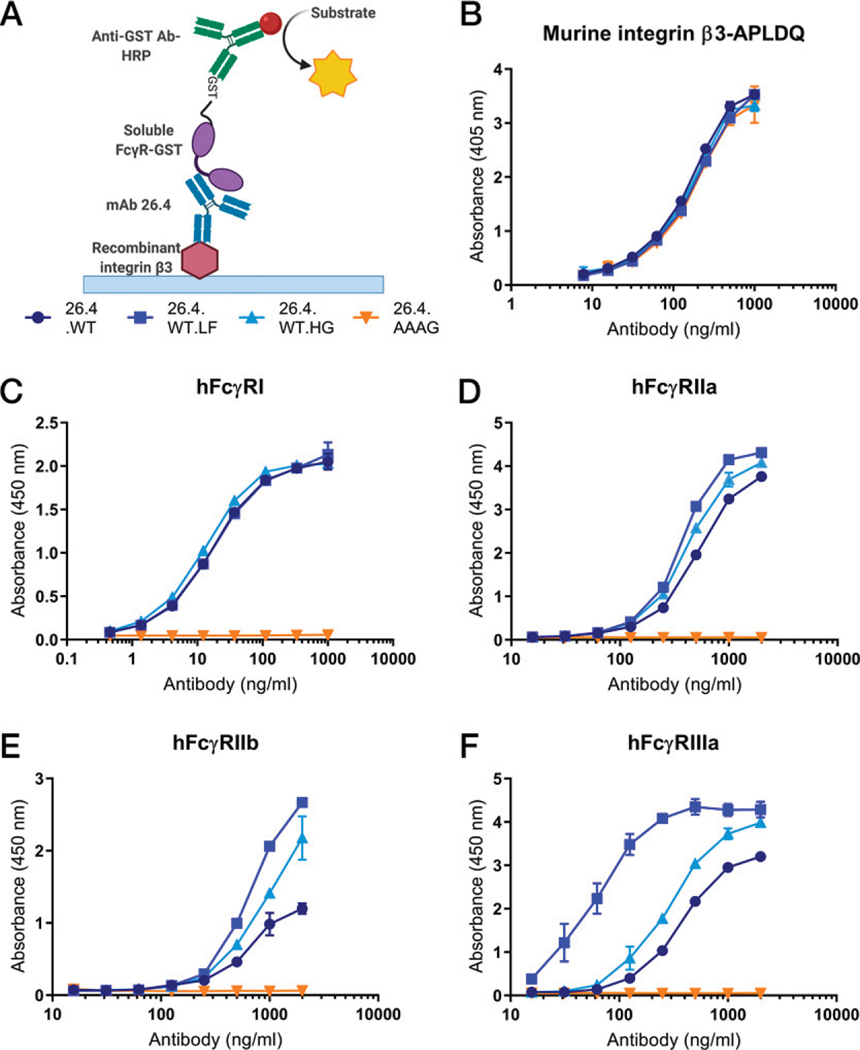
mAb 26.4 variants show distinct FcγR binding properties. (**A**) Schematic illustration of ELISA protocol for FcγR binding (created with BioRender.com). (**B**) Binding of titrated amounts of mAb 26.4 variants to recombinant integrin β3, a murine backbone, engineered with the HPA-1a epitope (APLDQ), coated in wells, and detected with an anti-human Fc Ab by ELISA. Each point is a mean of duplicates from one representative experiment out of two, and error bars indicate SD. (**C–F**) Binding of GST-tagged soluble recombinant human FcγRI (C), FcγRIIa-R131 (D), FcγRIIb (E), and FcγRIIIa-V158 (F) to titrated amounts of the mAb 26.4 variants captured on the integrin β3-APLDQ and detected with an HRP-conjugated anti-GST Ab. Each point is a mean of duplicates from one experiment, and error bars indicate SD. AAAG, LALA-N297A-PG.

After coating of the recombinant integrin β3 in ELISA wells, we added serial titrations of the purified mAb 26.4 variants followed by detection, which revealed that they bound the HPA-1a epitope equally well (Supplemental Fig. 1B, 1C). This was also the case when tested for binding to a recombinant version of the murine integrin β3 with the five amino acid substitutions (APLDQ), which introduce the human HPA-1a epitope (Fig. 2B). Thus, neither the AAAG substitutions nor alteration of posttranslational modification of the attached N-glycan structures affected Ag-Ab interactions.

Next, this ELISA setup was used to compare mAb binding with recombinant soluble versions of human FcγRs (Fig. 2A), which revealed that the AAAG substitutions completely eliminated binding to the high-affinity FcγRI (Fig. 2C), as well as the low-affinity FcγRIIa (Fig. 2D), FcγRIIb (Fig. 2E), and FcγRIIIa (Fig. 2F). In addition, reduced fucosylation of the N-glycans attached to N297 dramatically enhanced binding to FcγRIIIa (Fig. 2F), as expected. In terms of potential FcγR-mediated suppression of B cell activation (29, 30), 26.4.WT.LF showed increased binding to FcγRIIb compared with 26.4.WT (Fig. 2E), but also to FcγRIIa (Fig. 2D). The presence of galactose slightly enhanced binding to the low-affinity receptors compared with 26.4.WT (Fig. 2D-F).

**FIGURE 3.**
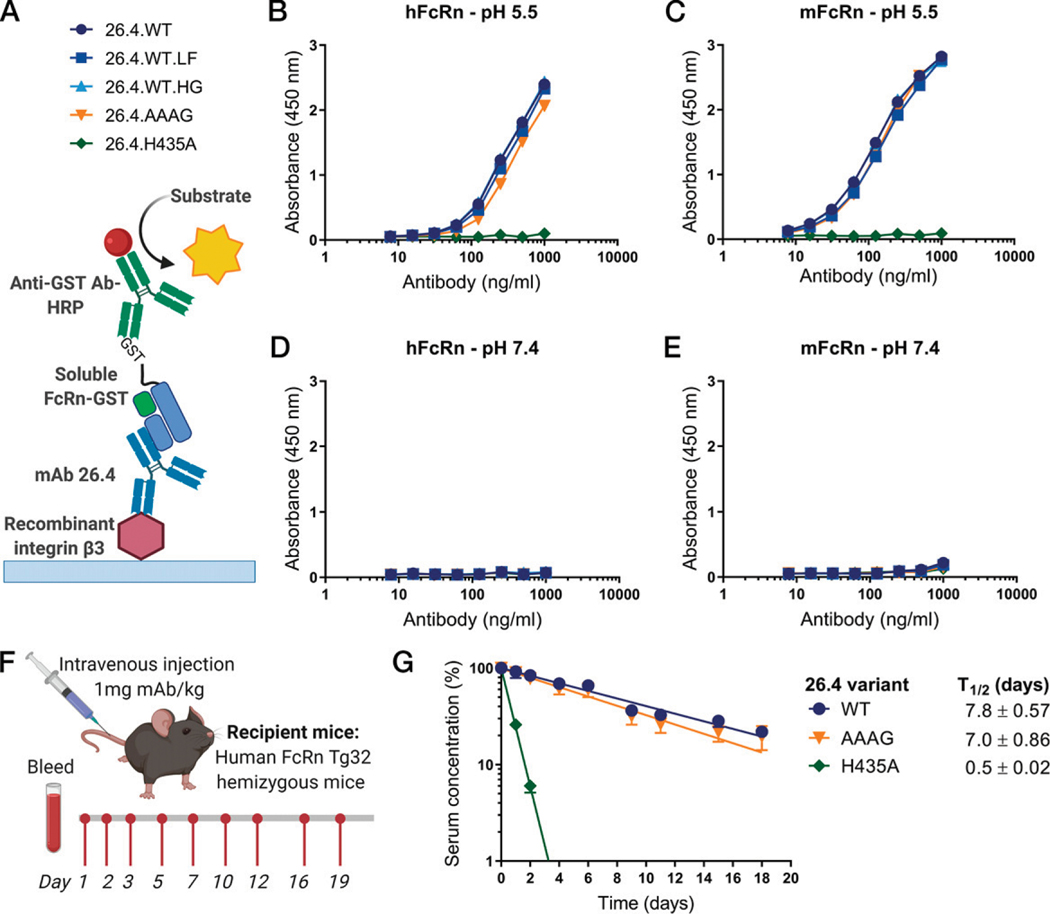
FcRn binding properties of the designed mAb 26.4 variants. (**A**) Schematic illustration of ELISA protocol for FcRn binding (created with BioRender.com). (**B–E**) Binding of recombinant GST-tagged soluble human (B and D) and mouse (C and E) FcRn to titrated amounts of the mAb 26.4 variants captured on recombinant integrin β3, a murine backbone, engineered with the HPA-1a epitope (APLDQ), at pH 5.5 (B and C) and pH 7.4 (D and E) measured by ELISA. Each point is a mean of duplicates from one representative experiment out of three, and error bars indicate SD. (**F**) Schematic illustration (created with BioRender.com) of the in vivo setup to measure the plasma half-lives of three mAb 26.4 variants injected (1 mg/kg) into human FcRn Tg32 hemizygous mice. Blood samples were drawn at days 1, 2, 3, 5, 7, 10, 12, 16, and 19 postadministration. (**G**) The presence of the mAb 26.4 variants in plasma derived from the Tg32 mice (*n* = 5 per Ab) measured by ELISA and analyzed using nonlinear regression analysis (GraphPad Prism 7) followed by plotting as percentage compared with day 1 (100%). Each point is a mean of duplicates, and error bars indicate SD. *t*_1/2_ is presented as days ± SD. AAAG, LALA-N297A-PG.

To verify the FcRn binding properties of the designed mAb 26.4 variants, we used the ELISA setup as described earlier, followed by adding recombinant forms of human and mouse FcRn (Fig. 3A), relevant for both the potential future in vivo use in humans and the current mouse model. In addition, a variant of 26.4 with the H435A substitution (26.4.H435A) was produced as a control Ab for reduced FcRn binding (15, 16). The results demonstrated equal binding of the 26.4 variants to the two receptor species at pH 5.5, except for 26.4.H435A (Fig. 3B, 3C), while none of them bound at pH 7.4 (Fig. 3D, 3E). Hence this is in line with the fact that the AAAG substitutions are structurally distal from the principal FcRn binding site (Fig. 1C) and, as such, are not expected to affect the plasma half-life. To address the latter, we used mice transgenic for human FcRn, which are the state-of-the-art model for plasma half-life determination of human IgG1 candidates (23). After i.v. administration of 26.4.WT and the AAAG variant, blood samples were collected over time, and the presence of the Abs in isolated plasma was quantified by ELISA (Fig. 3F). The results revealed a plasma half-life of 7–8 d for the mAbs; however, as a control for FcRn engagement, injection of 26.4.H435A gave rise to ~14-fold shorter plasma half-life (Fig. 3G).

### mAb 26.4 variants induce in vitro phagocytosis and in vivo platelet clearance

**FIGURE 4.**
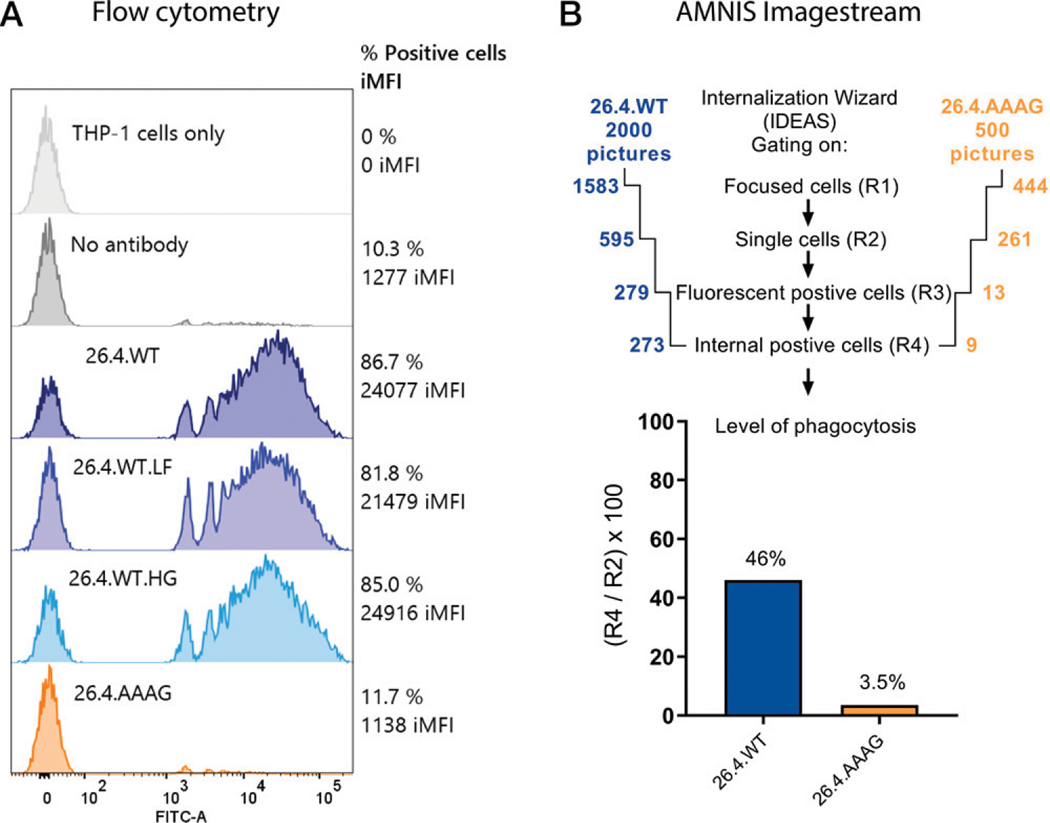
FcγR-mediated phagocytosis requires a fully functional Fc region of mAb 26.4. (**A**) Yellow-Green FluoSpheres coupled with recombinant integrin β3, a murine backbone, engineered with the HPA-1a epitope (APLDQ), coated with 10 μg/ml mAb 26.4 variants. Ab-dependent phagocytosis was measured in THP-1 cells after overnight incubation and measurement of 10,000 events in cell gate by flow cytometry. Data represent one experiment of multiple experiments with different time points and concentrations showing the same trend. THP-1 cells that were yellow-green fluorescent (intensity > 10^3^) are denoted with percentage, and iMFI was calculated with the formula: iMFI = percentage THP-1^+^ events × mean fluorescence intensity of positive events. (**B**) To confirm engulfment and not sticking of beads, 26.4.WT Ab (2000 pictures analyzed) and 26.4.AAAG Ab (500 pictures analyzed) were examined in a 1-h phagocytosis assay, measured by Amnis ImageStreamX Mk II (laser 488 0.3 mW and focus ×60; Luminex), and analyzed using the guide Internalization Wizard in the software IDEAS. Briefly, internalization was analyzed through multiple steps by gating on focused cells (R1), single cells (R2), fluorescent-positive cells (R3), and internal positive cells (R4). AAAG, LALA-N297A-PG.

For in vitro functional testing of the capacity of the Ab variants to induce phagocytosis, we measured uptake by THP-1 cells in a flow cytometric assay after binding to fluorescent beads with recombinant APLDQ Ag. Ab-dependent phagocytosis was efficient, independent of N-glycan difference, for all three mAb 26.4 WT variants (26.4.WT, 26.4.WT.LF, 26.4.WT.HG; Fig. 4A), in contrast with the binding differences in FcγRIIa (Fig. 2D) and FcγRIIIa (Fig. 2F) binding in ELISA. However, because phagocytosis is facilitated through multiple FcγRs on THP-1 cells (24, 31), the low FcγRIIIa expression in nondifferentiated monocyte-like THP-1 cells (32) may explain the poor correlation between phagocytic activity and FcγR binding efficacy for the Ab variants. To further support that the read-out in flow cytometry represents internalization of beads rather than mere surface binding, we also demonstrated internalization with 26.4.WT by imaging flow cytometry (Fig. 4B).

**FIGURE 5.**
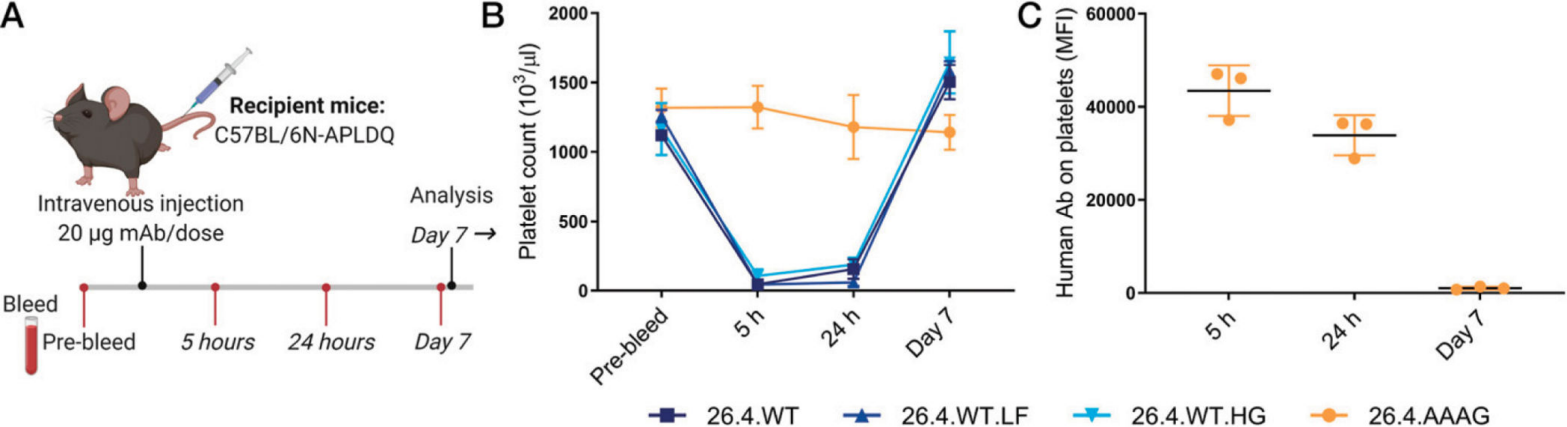
APLDQ mice injected with mAb 26.4 eliminate platelets from circulation. (**A**) Schematic illustration (created with BioRender.com) of C57BL/6N-APLDQ mice receiving mAb 26.4 injections i.v. following a prebleed. Blood samples were taken at 5 h, at 24 h, and after 1 wk. (**B**) Platelet count of APLDQ mice injected with 20 μg mAb 26.4 isoforms. (**C**) Platelets from APLDQ mice injected with 26.4.AAAG were isolated from blood samples and incubated with FITC-conjugated anti-human Ab to detect Abs on the platelet surface by flow cytometry, gated on platelets. MFI is plotted (*n* = 3) with mean and SD. AAAG, LALA-N297A-PG.

Because the prophylactic effect may depend on platelet clearance, 26.4.WT glycosylation variants (LF and HG) and 26.4.AAAG were injected into humanized APLDQ-expressing mice (Fig. 5A). 26.4.WT and glycosylation variants induced efficient platelet clearance in vivo, measured at 5 and 24 h after injection (Fig. 5B), with normalized platelet counts after 7 d. In contrast, 26.4.AAAG did not cause platelet clearance, although it was demonstrated to be bound on platelets at 5 and 24 h, but not detected after 7 d (Fig. 5C). To further investigate how the differential properties identified by in vitro studies translated into immunosuppression in the preclinical model, we included 26.4.WT and 26.4.AAAG in subsequent experiments.

### Administration of mAb 26.4 prevents immunization against HPA-1a in vivo

**FIGURE 6.**
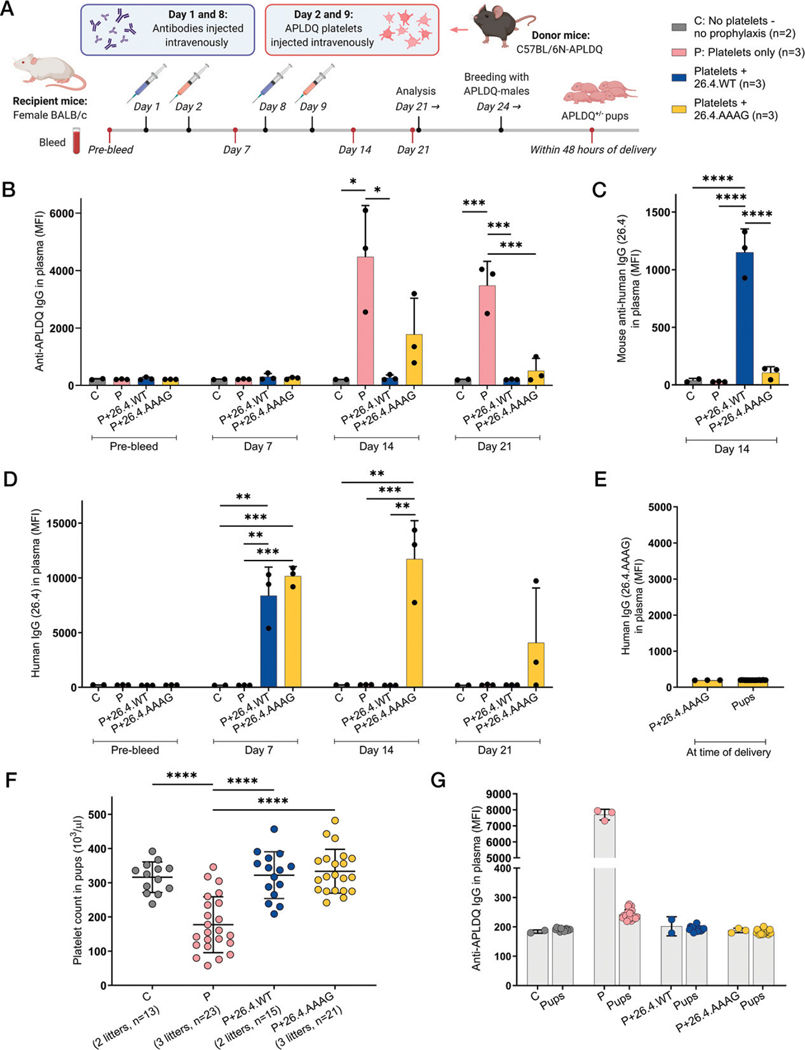
mAb 26.4 isoforms induce AMIS in FNAIT mouse model. (**A**) Schematic illustration (created with BioRender.com) of the FNAIT mouse model where BALB/c mice receive Ab injections (20 μg/dose) at days 1 and 8, and APLDQ platelets (10^8^/dose) isolated from C57BL/6N-APLDQ mice on days 2 and 9. Mice were bled 1 week prior to experiment start and at days 7, 14, and 21. After plasma analysis for anti-APLDQ mouse IgG Abs (**B**), MAHAs (**C**), and human IgG Abs (**D**), the mice (*n* = 2–3) were bred with APLDQ males and newborn pups measured for the presence of 26.4.AAAG in plasma (**E**), platelet count (**F**), and anti-APLDQ Ab levels (**G**). (B and C) APLDQ platelets were isolated and incubated with plasma samples followed by detection using FITC-conjugated anti-mouse IgG Ab (B) or anti-human IgG Ab (D) measured by flow cytometry gated on platelets. (C) MAHAs were detected from plasma on day 14 using Dynabeads coated with mAb 26.4 and detection using Alexa Fluor 647–conjugated goat anti-mouse Ab measured by flow cytometry gated on a single bead population. (E–G) Blood samples were collected from newborn pups within 48 h of delivery, and presence of 26.4.AAAG Abs was measured in plasma from pups and moms in the 26.4.AAAG group (E), platelet count (F) was measured using ScilVet ABC (scil animal care company), and anti-APLDQ Abs (G) measured in both pups and moms. MFI is plotted with mean and SD. Statistical data were analyzed with one-way ANOVA and Tukey multiple comparison test of means against mean of immunized mice (*p ≤ 0.05, **p ≤ 0.01, ***p ≤ 0.001, ****p ≤ 0.0001). Nonsignificant comparisons were not shown. AAAG, LALA-N297A-PG.

By the use of an FNAIT mouse model where murine APLDQ platelets lead to the generation of anti-APLDQ Abs when injected into BALB/c mice, the prophylactic potential of the two mAb 26.4 variants was studied by injecting 20 μg mAb 24 h prior to platelet transfusion (Fig. 6A). Efficient endogenous anti-APLDQ Ab responses were seen at days 14 and 21 in mice challenged with platelets only, while prophylactic treatment with 26.4.WT completely prevented this immune response (Fig. 6B). Mice injected with 26.4.AAAG showed detectable anti-APLDQ Abs at day 14, but the mean level was significantly lower than in the immunization control group at day 21 (*p* = 0.0008). The administered Abs were both detectable in plasma at day 7 (Fig. 6D), while only 26.4.AAAG was present in plasma at days 14 and 21. Notably, generation of MAHAs was observed in plasma from 26.4.WT-injected mice at day 14, but interestingly, not against 26.4.AAAG (Fig. 6C). All plasma samples showed no or very low Ab reactivity against platelets from BALB/c and C57BL/6N mice (Supplemental Fig. 3).

To assess the effect on clinical outcome, we bred the preimmunized female mice from the prophylaxis experiment with APLDQ males. No remaining 26.4.AAAG Abs were detected in plasma from pups or moms in the 26.4.AAAG group (Fig. 6E). Pups from immunized mice showed significantly reduced platelet counts (*p* < 0.0001), whereas pups from Ab-treated females all presented normal platelet counts (Fig. 6F), even though detectable levels of anti-APLDQ Abs were seen at day 14 in 26.4.AAAG-treated mice. When measuring the anti-APLDQ Ab levels in plasma of both mothers and pups, only pups from immunized mothers showed the presence of anti-APLDQ Abs (Fig. 6G). Taken together, these data provide the proof of principle that mAb 26.4 has potential as a specific drug for FNAIT prophylaxis by hindering immunization in BALB/c mice against transfused APLDQ platelets carrying the HPA-1a epitope. Prophylactic administration of the mAb 26.4 WT, and to some extent the mAb 26.4 effector silent variant, prior to platelet transfusion resulted in reduced alloimmunization in challenged mice and normal platelet counts in neonates.

## DISCUSSION

The purpose of the study was to explore the prophylactic potential of a panel of recombinant isoforms of mAb 26.4 to prevent HPA-1a immunization in a recently developed preclinical FNAIT mouse model (12). The HPA-1a homologous APLDQ epitope has been shown to be a target for several polyclonal alloanti-HPA-1a sera, and several, but not all, monoclonal anti-HPA-1a Abs tested (33). Importantly, mAb 26.4 binds efficiently to the APLDQ epitope while being nonreactive with WT murine platelets, making this preclinical model highly suitable for in vivo therapeutic (12) and prophylactic evaluation. Indeed, mAb 26.4 showed great potential as an FNAIT prophylaxis by inducing AMIS in BALB/c mice transfused with APLDQ platelets, with no sign of thrombocytopenia in subsequent neonates.

A requirement for a prophylactic mAb is that it exclusively binds HPA-1a, thereby eliminating any harmful interaction with maternal HPA-1bb platelets. However, the optimal design of a prophylactic recombinant mAb for prevention of HPA-1a alloimmunization in humans may also depend on the planned timing of administration. For a tentative postpartum treatment strategy, the prophylactic effect may well be immediate and without the need for a long plasma half-life. In contrast, antenatal prophylactic mAb administration requires attention to the risk of potential pathogenic effect on placenta and fetal platelets. With this in mind, we designed mAb 26.4 variants as prophylactic candidates, with and without effector functions.

Although the plasma half-life of IgG1 is 3 wk on average in humans, clinical studies have shown that the half-lives of IgG1 mAbs vary greatly, between 6 and 32 d, despite the fact that they all contain the same Fc (34). One reason for this is that the composition of the variable regions may affect cellular handling, and subsequently pharmacokinetics. Such effects may be dependent on the interaction with the half-life regulator FcRn (35, 36), which is a cellular receptor that interacts in a strictly pH-dependent manner at the CH2-CH3 elbow region of the IgG Fc (37–39). Specifically, this mode of binding is regulated by protonation of histidine residues (H310 and H435) (Fig. 1C), allowing strong binding at acidic pH (5.0–6.0) and no binding or release at neutral pH. Importantly, this pH-dependent binding is also required for FcRn-mediated mother-to-fetus transport across the placenta (40–44). The plasma half-lives of 26.4.WT and 26.4.AAAG were determined to be 7–8 d in the human FcRn in vivo mouse model. This is a relatively long plasma half-life in this mouse model, which predicts that the pharmacokinetics in humans should be favorable.

The mechanisms that likely contribute to AMIS have been reviewed for anti-D Abs against RhD immunization (29, 30). Briefly, by the Ag clearance or destruction theory, the particles containing Ags are rapidly removed through Fc-mediated mechanisms, such as Ab-dependent cellular phagocytosis by splenic macrophages or Ab-dependent cellular cytotoxicity. In the B cell inhibition theory, the BCR binds the Ag concurrently with the inhibitory FcγRIIb on the B cell binding to the Fc region of Abs bound to the same particle (coinhibition), thus hindering further proliferation, maturation, and Ab secretion of the B cell. These two theories are the most likely mechanisms behind AMIS, but also epitope masking and Ag modulations may contribute. Accordingly, Ag clearance in this model is represented as platelet clearance, which was efficiently induced by mAb 26.4.WT variants when injected into APLDQ mice, in line with recent data on this model (12). In contrast, the effector silent 26.4.AAAG did not induce in vivo platelet clearance, although present on the platelet surface.

In the immunization experiment with prophylactic administration of mAb 26.4, the generation of murine anti-APLDQ Abs was prevented by 26.4.WT but also significantly reduced with 26.4.AAAG. Because this engineered isoform lacks effector functions, the less efficient prevention by 26.4.AAAG is likely due to epitope masking only, as seen in the platelet clearance experiment, and less efficient removal of either Ab or bound transfused platelets. Thus, the prophylactic effect of 26.4.AAAG demonstrates that rapid platelet clearance is not the sole mechanism of action at the relatively high dose (20 mg Abs/mouse) used in this study. For RBC alloimmunization, there are indications that AMIS most often confers Ag-specific prevention (45), but on high-density Ag expression also non-epitope-specific prevention to neighboring Ags, tentatively by both epitope masking and steric hindrance (46). However, because no or very low Ab responses were detected to other alloantigens on C57BL/6N platelets in all groups, our results could not indicate whether the protection was solely epitope specific (Supplemental Fig. 3).

In subsequent breeding experiments, litters from mice that received Ab prophylaxis had normal platelet counts, including those in the 26.4.AAAG group that had only partially prevented immunization. Circulating 26.4.AAAG Abs in females during pregnancy could potentially be protective against fetal platelet clearance by pathogenic maternal anti-APLDQ Abs. However, the remaining low levels of 26.4.AAAG Abs at the time of breeding and the notion that no 26.4.AAAG Abs were detected in plasma from either pups or mothers at time of delivery makes it unlikely that these Abs have masked epitopes on fetal platelets to the level of protection.

Plasma from mice given both APLDQ platelets and prophylactic Abs, may eventually contain prophylactic IgG in addition to endogenous murine anti-APLDQ IgG. Even with use of species-specific secondary reagents for IgG detection, the human prophylactic Ab and the murine anti-APLDQ Abs may compete for the same epitope on the APLDQ test platelets and thus might impact the read-out of Ab detection. However, this is not likely impacting our data because both anti-APLDQ Abs and 26.4.AAAG Abs plasma levels were reduced from day 14 to 21 in the 26.4.AAAG mice. Further, it is known that human Abs can trigger generation of MAHA (anti-drug Abs) in preclinical models. Interestingly, the 26.4.AAAG variant raised little MAHA response, which otherwise could negatively affect its protective effect.

The proof of concept for AMIS, relevant for FNAIT, was previously demonstrated as dampened immune response by injecting murine anti-integrin β3 Abs into β3-knockout mice after transfusion of WT platelets (47). In addition, human polyclonal anti-HPA-1a Abs from alloimmunized women or murine mAb SZ21 also induced AMIS in the same model when transfusing human HPA-1a^1^ platelets (47). However, AMIS was also induced in knockout mouse models for activating or inhibitory FcγR using intact Ab, but also with the use of Fab fragments, indicating that AMIS can occur through Fc- and FcγR-independent pathways (48). Although convincingly demonstrating AMIS in the earlier models, their intrinsic drawback is the lack of ability to include the specific epitope relevant to test human Ab candidates on otherwise normal murine platelets.

In our model, APLDQ immunization mimicking HPA-1a alloimmunization can be achieved by either i.p. injections with adjuvant or i.v. by tail-vein injections. In the literature, the administration protocols of prophylaxis in AMIS/clearance experiments varies from protocols where injection occurs prior to administering a dose of immunizing agent, to ex vivo incubation of Ag and Ab prior to injection, to simultaneous administration of Ag and Ab, or to Ab administration shortly after Ag administration (47, 49–51). Here, we administered the prophylactic Abs 24 h prior to platelet transfusion, to ensure presence of prophylactic Abs in circulation. By using tail-vein administration without adjuvant, we mimic Ag exposure through fetomaternal hemorrhage during pregnancy or in connection with delivery. Notably, by using this i.v. immunization protocol, the Ab responses did not reach levels achieved with i.p. injections with adjuvant, and accordingly, FNAIT was less severe in affected pups (12). We currently have no data on the fate of the transfused APLDQ platelets with regard to the persistence in circulation and anatomical location for destruction in vivo after administration of the different prophylactic Ab variants. Future in vivo studies on this may give further insight into the preventive mechanism. The mAb 26.4 has previously been reported not to induce aggregation of HPA-1aa platelets, in line with other anti-HPA-1a Abs (11, 52). A similar impact on murine APLDQ platelets was not investigated in this study because the transfused platelets served mainly as Ag in our model. However, given that a change in integrin β3 because of mAb 26.4 binding likely depends on the Ab-epitope interaction, all Ab isoforms should exert the same effect. We did not find any indications that the effector silent mAb 26.4.AAAG caused platelet aggregation in vivo in APLDQ mice, because opsonized platelets were still circulating after 24 h.

From a translational perspective, the human HPA-1a epitope on αIIbβ3 is in addition to being abundantly expressed on platelets, also present on the αvβ3 on placental syncytiotrophoblasts (53) and endothelial cells (54). Importantly, maternal anti-HPA-1a Ab selectively reactive with αVβ3 complexes on endothelial cells has been reported to be more strongly associated with FNAIT-associated intracranial hemorrhage than those selectively reactive with αIIbβ3 (54). Relevant to this, mAb 26.4 binds independently of the α subunit (11) and also to the β3 subunit alone (Supplemental Fig. 1B). For postpartum prophylactic administration of mAb 26.4, this may be appropriate, while for antenatal prophylactic use, an effector silent isoform therefore could be preferential to avoid harmful effects on placenta, endothelial cells, or platelets of fetal origin. However, future dosage experiments may conceivably balance the dose required for prevention and undesirable interaction with fetal Ag for antenatal use. In theory, a variant with abolished binding to FcRn and thus limited transport into fetal circulation would limit the risk of pathogenic effect in the fetus. However, because the IgG1 half-life in circulation and transplacental transport are both dependent on FcRn, such a prophylactic variant would require a design that could allow dissection of these two parameters.

In conclusion, we have successfully shown that variants of the mAb 26.4 can prophylactically prevent FNAIT in the APLDQ pre-clinical model, supporting further studies toward use in humans.

## Supplementary Material

Supplementary Material

## Abbreviations used in this article:

AMIS: Ab-mediated immune suppression
AP: alkaline phosphatase
EIA: enzyme immunoassay
FNAIT: fetal and neonatal alloimmune thrombocytopenia
HG: high galactose
HPA: human platelet Ag
iMFI: integrated mean fluorescence intensity
LF: low fucose
MAHA: mouse anti-human Ab
MFI: median fluorescence intensity
RT: room temperature
WT: wild-type
