# Supplementary material for: Prevention of Fetal/Neonatal Alloimmune Thrombocytopenia in Mice: Biochemical and Cell Biological Characterization of Isoforms of a Human Monoclonal Antibody
Source: Immunohorizons. Author manuscript; Available in PMC 2023 Apr 12. (PMC10094187; doi:10.4049/immunohorizons.2100097)
Supplement: Supplementary Material [file NIHMS1861763-supplement-Supplementary_Material.pdf]

Supplementary materials

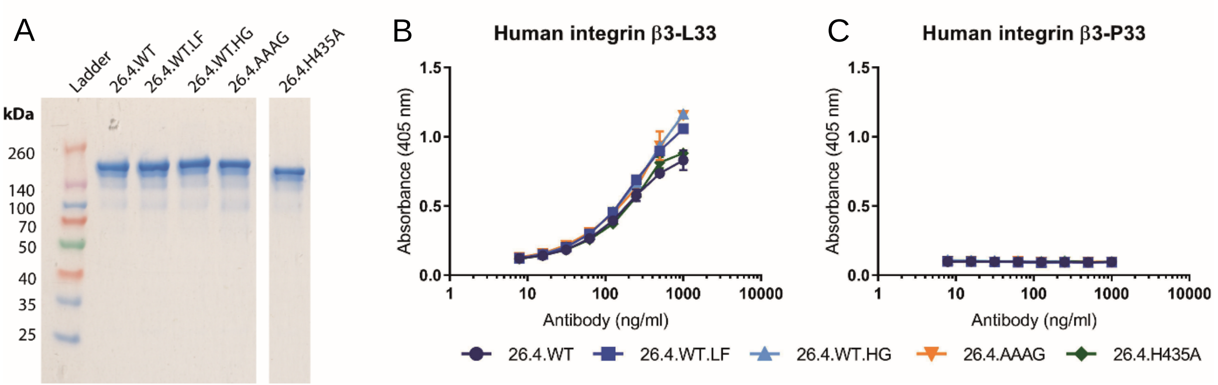

SUPPLEMENTARY FIGURE S1. Recombinant Fc region variants of mAb 26.4 bind specifically to HPA-1a. *Anti-HPA-1a 26.4 IgG1 isoforms produced recombinantly with modifications. (**A**) Purified mAb 26.4 variants were run on a non-reducing SDS-PAGE, and all migrated with expected molecular weights (150 kDa) and purity. (**B**-**C**) Binding of titrated amounts of mAb 26.4 variants to recombinant soluble β3 integrins variants L33 (**B**) and P33 (**C**) coated in ELISA wells (absorbance 405nm). Each point is a mean of duplicates from one representative experiment out of two, and error bars indicate SD.*

Downloaded from http://journals.aai.org/immunohorizons/article-supplement/234066/pdf/ih_2100097_supplemental_1/ by guest on 03 April 2023

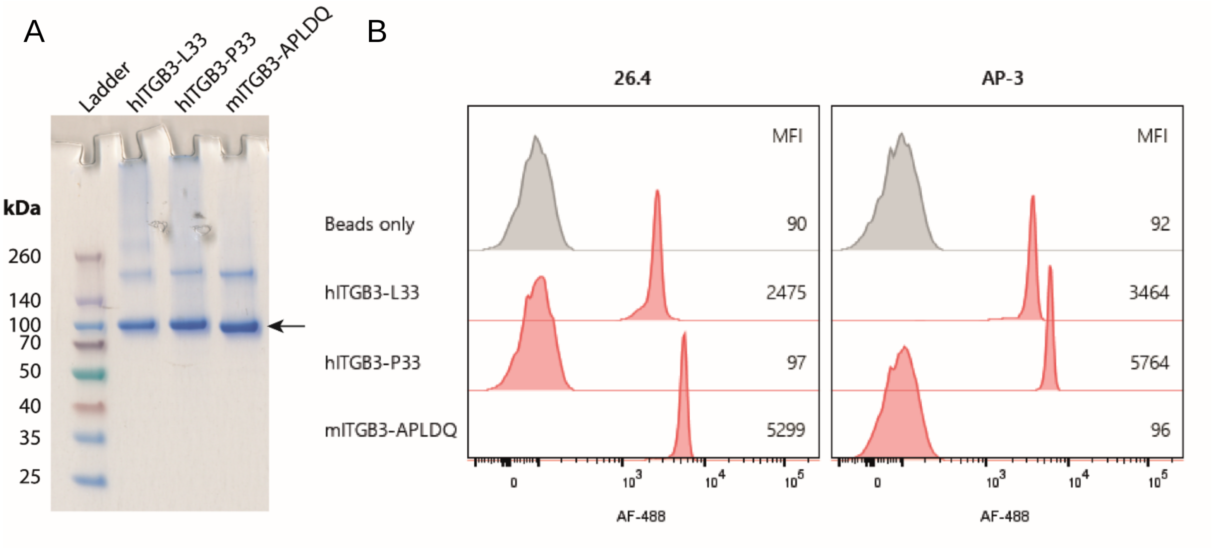

SUPPLEMENTARY FIGURE S2. mAb 26.4 bind soluble recombinant integrin β3, a murine backbone, engineered with the HPA-1a epitope (APLDQ). *The extracellular domains of ITGB3 with a 6xhis-tag and Twin-Strep-tag were produced as soluble proteins in insect cells using the baculovirus system. Constructs for human integrin β3-L33 (hITGB3-L33), human integrin β3-P33 (hITGB3-P33) and murine integrin β3 engineered with the HPA-1a epitope (mITGB3-APLDQ) were produced. (**A**) Recominant integrin β3 proteins were run on a non-reducing SDS-PAGE showing the presence of proteins around 100 kDa (arrow) similar to a teoretical size of approximately 85 kDa. (**B**) Binding of recombinant integrin β3 proteins, captured on Dynabeads™ His-Tag Isolation and Pulldown, to anti-integrin β3 Abs detected by flow cytometry. Expectedly, AP-3 (AF-488), a mouse anti-human Integrin β3 Ab, bound both human integrins-L33 and -P33 (hITGB3-L33/P33), and 26.4 (AF-488) bound recombinant proteins comprising the HPA-1a epitope (L33 and APLDQ variants: hITGB3-L33 and mITGB3-APLDQ). 10000 events were collected. MFI=median fluorescence intensity.*

Downloaded from http://journals.aai.org/immunohorizons/article-supplement/234066/pdf/ih_2100097_supplemental_1/ by guest on 03 April 2023

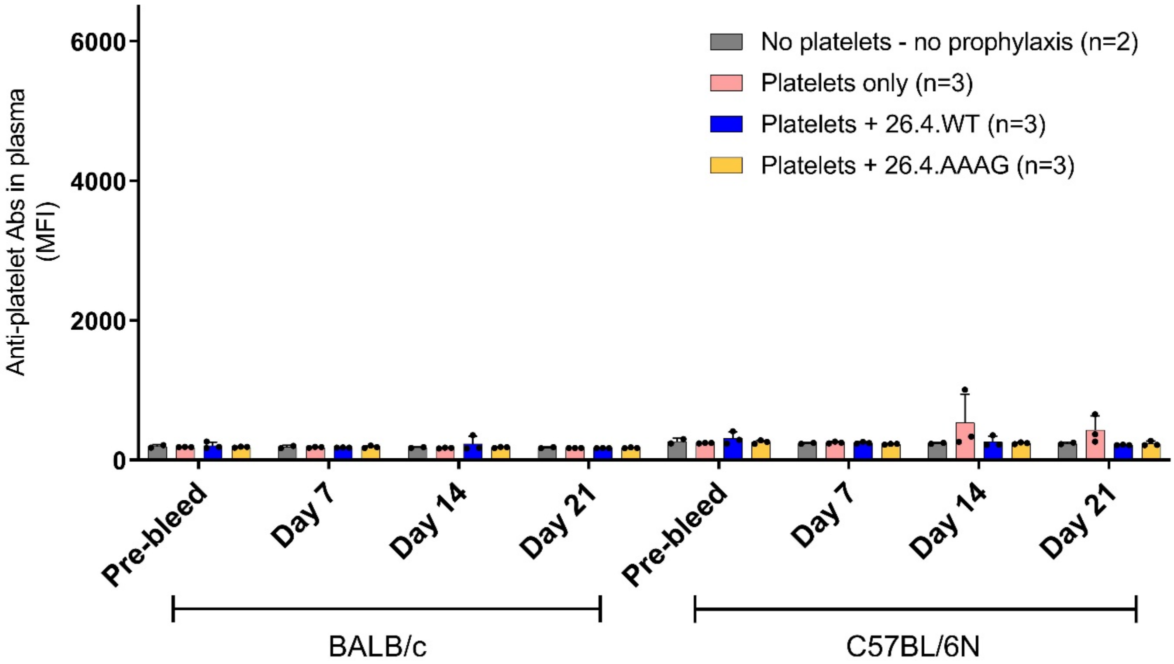

SUPPLEMENTARY FIGURE S3. No or negligible binding of mAb 26.4 variants to control murine platelets from AMIS experiments. *Platelets isolated from BALB/c and C57BL/6N mice were isolated and incubated with plasma samples followed by detection using FITC-conjugated anti-mouse IgG Ab and analyzed flow cytometry. Median fluorescence intensity (MFI) is plotted with mean and SD.*

Downloaded from http://journals.aai.org/immunohorizons/article-supplement/234066/pdf/ih_2100097_supplemental_1/ by guest on 03 April 2023
